# Sulfasalazine-Induced Drug Reaction With Eosinophilia and Systemic Symptoms (DRESS) Syndrome in a Patient With Rheumatoid Arthritis

**DOI:** 10.7759/cureus.91527

**Published:** 2025-09-03

**Authors:** Ayesha Babar, Abir Aijaz, Abdul Bhat, Amit Badshah, Zagham Hammad

**Affiliations:** 1 Acute Medicine, Weston General Hospital, University Hospitals Bristol and Weston, Weston-super-Mare, GBR; 2 Acute and General Internal Medicine, University Hospitals Bristol and Weston, Weston-super-Mare, GBR; 3 Acute Medicine, University Hospitals Bristol and Weston, Weston-super-Mare, GBR

**Keywords:** drug reaction with eosinophilia and systemic symptoms (dress), skin lesions, sulfa drug, sulfasalazine, sulfasalazine-induced hypersensitivity syndrome

## Abstract

Because of its non-specific presentation and slow start, drug reaction with eosinophilia and systemic symptoms (DRESS) syndrome is a severe and frequently underdiagnosed hypersensitivity reaction. In this case study, we discuss a 62-year-old lady who had been using sulfasalazine treatment for rheumatoid arthritis and presented to the emergency room with an extensive erythematous rash that was particularly noticeable on her lower limbs. She was found to have eyelid puffiness, nausea, and chills as well. Based on established diagnostic criteria, imaging scans, laboratory data, and clinical indicators, DRESS syndrome was diagnosed. This case emphasizes how important it is to identify DRESS syndrome early and be more clinically vigilant by adapting a timely multidisciplinary strategy to manage it successfully and avoid major systemic consequences.

## Introduction

Drug reaction with eosinophilia and systemic symptoms (DRESS) syndrome is a rare, severe, and potentially life-threatening drug-induced hypersensitivity reaction characterized by a constellation of clinical features, including fever, widespread cutaneous eruptions, hematologic abnormalities such as eosinophilia, and multi-organ involvement [1].

Diagnosis is often challenging due to its delayed onset, typically two to eight weeks after exposure to the causative agent, and nonspecific early symptoms, such as fever and malaise [2]. The rash, often beginning on the face, trunk, and upper limbs, may progress from a morbilliform or urticarial pattern to erythroderma or culminate in exfoliative dermatitis [3]. Facial edema and mucosal changes, such as cheilitis or pharyngeal erythema, are also frequent. The pathogenesis involves a complex interplay of genetic predisposition, drug-specific immune responses, and viral reactivation, particularly human herpesvirus 6 (HHV-6). Hepatic involvement is most common, ranging from mild enzyme elevations to fulminant hepatic failure, though the cardiac, renal, pulmonary, and hematologic systems may also be affected [4].

The RegiSCAR scoring system assists in classifying the likelihood of DRESS syndrome using clinical, laboratory, and histopathological criteria. Effective management relies on early recognition, immediate withdrawal of the offending agent, systemic corticosteroids, and supportive care. This case contributes to the growing literature on DRESS and emphasizes the importance of timely diagnosis and a multidisciplinary approach to management [5].

## Case presentation

A 62-year-old woman presented to the emergency department on July 5, 2025, with a one-day history of a worsening generalized rash. The rash was erythematous, maculopapular, and involved her trunk, arms, and legs. She had recently restarted sulfasalazine for rheumatoid arthritis and developed symptoms including high-grade fever, eyelid swelling, nausea, chills, and bronchospasm. Notably, she had experienced a similar rash episode a few days earlier after reaching the full prescribed dose of sulfasalazine on June 30 while in Turkey. She was treated and recovered, but resumed the medication on July 4, after which the rash returned more severely. She denied any prior history of allergies or adverse reactions to sulfa-containing drugs. Her medical history included rheumatoid arthritis, hypertension, and type 2 diabetes mellitus, with no recent infections.

**Figure 1:**
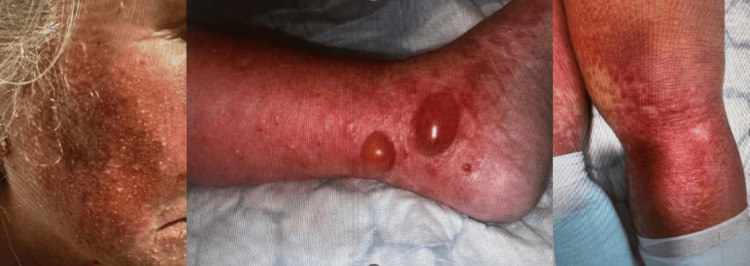
Extensive rash involving the face and limbs.

On examination, she appeared ill, febrile, and visibly uncomfortable due to extensive skin involvement. She was alert and oriented. Her vital signs were as follows: temperature at 102°F; pulse rate of 104 bpm; respiratory rate of 24 breaths/min; blood pressure at 130/85 mmHg; and oxygen saturation of 94% on room air. The skin exam revealed a widespread, non-blanching, itchy, warm maculopapular rash sparing the mucous membranes (Figure 1). There was noticeable facial puffiness and periorbital edema.

No pallor, jaundice, or cyanosis was noted. Bilateral cervical and axillary lymph nodes were palpable: small, firm, mobile, and non-tender. Oral mucosa appeared moist without lesions, though mild dehydration was evident from dry lips and tongue.

Respiratory examination showed a central trachea, symmetrical chest expansion, and tachypnea with accessory muscle use. Auscultation revealed bilateral vesicular breath sounds with scattered wheezing, while percussion was resonant bilaterally. There were no crackles or signs of consolidation. These findings suggested a systemic hypersensitivity reaction to sulfasalazine, with significant skin and respiratory involvement.

Bronchospasm was relieved by nebulization with salbutamol, and the patient was hydrated with intravenous fluids. Further treatment included prednisolone 40 mg and a topical emollient with proton pump inhibitors for gastroprotection. Treatment included a multidisciplinary approach involving emergency, dermatology, and general medicine with timely rheumatology input. Investigations are presented in Tables 1, 2.

**Table 1:** Table summarizing skin biopsy findings. PCR: polymerase chain reaction.

Investigation	Sample details	Findings/results
Histopathology & immunofluorescence	4 mm punch biopsy from the right arm	Microscopy revealed a dense, superficial, and deep perivascular lymphocytic infiltrate with prominent eosinophils and interface vacuolar changes. Mild spongiosis was noted. Immunofluorescence: Direct immunofluorescence was negative for IgG, IgM, IgA, C3, and fibrinogen.
Viral PCR	Swab from the blister site	Herpes simplex virus 1 (PCR): Negative. Herpes simplex virus 2 (PCR): Negative.
Wound Swab (Culture & Sensitivity)	Wound swab from the hand	Culture: Scanty growth of *Staphylococcus aureus*. Sensitivity: Sensitive to flucloxacillin and clarithromycin; resistant to doxycycline.

**Table 2:** Investigations. RBC: red blood cells; MCV: mean corpuscular volume; MCH: mean corpuscular hemoglobin; MCHC: mean corpuscular hemoglobin concentration; RDW: red cell distribution width; APTT: activated partial thromboplastin time; eGFR: estimated glomerular filtration rate; INR: international normalized ratio.

Test	Day 1	Repeat investigations
C-reactive protein (CRP)	23 mg/L	12 mg/L
White cell count (WBC)	8.50 ×10⁹/L	17.60 ×10⁹/L ↑
RBC	3.26 ×10¹²/L ↓	3.29 ×10¹²/L ↓
Hemoglobin	117 g/L ↓	111 g/L ↓
Hematocrit	0.326 L/L ↓	0.327 L/L ↓
MCV	100.0 fL	99.4 fL
MCH	35.9 pg ↑	33.6 pg
MCHC	359 g/L ↑	338 g/L
Platelets	227 ×10⁹/L	338 ×10⁹/L
RDW	12.8 %	13.1 %
Neutrophils	6.13 ×10⁹/L	13.1 ×10⁹/L ↑
Lymphocytes	1.48 ×10⁹/L ↓	7.74 ×10⁹/L
Monocytes	1.14 ×10⁹/L	4.93 ×10⁹/L ↑
Eosinophils	0.65 ×10⁹/L ↑	1.76 ×10⁹/L ↑
Basophils	0.09 ×10⁹/L	–
Myelocytes	–	2.99 ×10⁹/L ↑
Prothrombin time	–	10.5 s
APTT	–	22.5 s ↓ (low)
INR	–	0.9
APTT ratio	–	<0.75
Sodium	137 mmol/L	–
Potassium	5.1 mmol/L	–
Urea	6.1 mmol/L	–
Creatinine	81 μmol/L	–
eGFR (CKD-EPI)	67 mL/min ↓	–

## Discussion

DRESS syndrome is a rare but potentially fatal adverse drug reaction, with a mortality rate of approximately 10% [1]. This case presents a classical and severe manifestation of the syndrome, which is frequently associated with sulfasalazine, a commonly prescribed disease-modifying antirheumatic drug. The patient's clinical picture, including high-grade fever, diffuse maculopapular rash sparing mucosal membranes, facial edema, lymphadenopathy, and systemic complications including bronchospasm, satisfies established diagnostic criteria. The rapid recurrence of symptoms upon re-challenge with sulfasalazine strongly supports a hypersensitivity mechanism.

The underlying pathogenesis of DRESS is complex and not fully understood, but it involves a drug-specific immune response often linked to certain human leukocyte antigen (HLA) alleles, resulting in robust T-cell activation [6]. This cascade culminates in a cytokine storm, with elevated IL-5 levels contributing to the hallmark eosinophilia. Reactivation of latent herpesviruses, particularly HHV-6, plays a central role by prolonging the inflammatory process and worsening disease severity. Sulfasalazine, a sulfonamide derivative, is among the most well-recognized triggers for DRESS syndrome.

Diagnosing DRESS is challenging due to its clinical resemblance to infectious, autoimmune, or dermatologic conditions. A high index of clinical suspicion is essential, especially given the syndrome’s delayed onset, typically two to eight weeks after initiating the offending drug. Effective management hinges on immediate and permanent discontinuation of the causative agent and early administration of systemic corticosteroids, which remain the cornerstone of treatment [7]. Steroids are usually tapered gradually to prevent relapse. In steroid-refractory cases, immunosuppressants such as cyclosporine or intravenous immunoglobulin (IVIG) may be considered. The case underscores the importance of vigilant monitoring and a multidisciplinary approach to mitigate the associated morbidity and mortality.

DRESS is rare, occurring in one in 1,000-10,000 drug exposures globally. Hospital-based studies suggest an incidence ranging from 2.2 to 40 per 100,000 hospitalized patients, with increased prevalence among women and individuals of Black descent. Regional variations exist, with rates reported at 9.6 per 100,000 inpatients in Thailand and 2.18 per 100,000 in the United States [8]. The fatality rate stands at around 3.8%, often due to complications like hepatic necrosis. In countries like Pakistan, underreporting and low clinical awareness result in a lack of comprehensive prevalence data.

Clinically, DRESS typically manifests two to six weeks after drug exposure with a widespread morbilliform rash beginning on the face and upper trunk. Facial edema, seen in about 25% of cases, is considered pathognomonic. Severe forms can evolve into exfoliative erythroderma. The triad of fever (>38.5°C), lymphadenopathy, and multi-organ involvement is central to diagnosis. Lymphadenopathy is present in 70-75% of cases, often involving multiple nodes over 2 cm in diameter. The systemic involvement contributes to the relatively high mortality of 5-10%. Hematologic findings include eosinophilia (≥1.5 × 10⁹/μL), atypical lymphocytosis, and leukocytosis, although eosinophilia may be absent in up to 10% of cases [9,10]. Thrombocytopenia, when present, signals poor prognosis.

Hepatic involvement is the most frequent systemic complication, occurring in 60-90% of cases and marked by elevated transaminases [11]. Severe liver involvement can lead to fulminant hepatic failure. Renal complications, affecting 10-20% of patients, often present as interstitial nephritis with elevated creatinine and proteinuria. Cardiac and pulmonary involvement, though less common, can be life-threatening.

Diagnosis involves a comprehensive clinical assessment and a range of laboratory tests, including CBC with differential, liver and renal function tests, and serologies for viral reactivation, especially HHV-6. Imaging, such as chest X-rays or ultrasounds, and tests like antinuclear antibody (ANA) and viral serologies help rule out differentials [12]. Skin biopsies may support, but are not definitive for diagnosis. Tools like the RegiSCAR scoring system aid in confirming DRESS and evaluating disease severity [13].

## Conclusions

This case of sulfasalazine-induced DRESS syndrome in a 62-year-old woman highlights the insidious and non-specific onset of a potentially life-threatening hypersensitivity reaction. Initially presenting with symptoms such as widespread erythematous rash, facial edema, lymphadenopathy, fever, and bronchospasm, the condition escalated rapidly upon re-exposure to the drug. DRESS syndrome poses a significant diagnostic challenge due to its delayed onset and its potential for severe multi-organ involvement, including the liver, kidneys, lungs, and heart, which can result in fatal outcomes. Prompt identification is critical, as early withdrawal of the offending agent and initiation of systemic corticosteroids are the mainstays of effective treatment. This case emphasizes the need for high clinical suspicion, especially in patients using sulfasalazine for conditions such as rheumatoid arthritis. A multidisciplinary approach is vital to managing systemic complications and improving patient outcomes. Clinicians must remain alert to the risk of recurrence with re-exposure, as DRESS syndrome can reappear more rapidly and with increased severity. Awareness of the syndrome’s latency period and the risks associated with re-challenge is essential for early diagnosis and prevention of severe complications.
